# Blood transfer devices for malaria rapid diagnostic tests: evaluation of accuracy, safety and ease of use

**DOI:** 10.1186/1475-2875-10-30

**Published:** 2011-02-08

**Authors:** Heidi Hopkins, Wellington Oyibo, Jennifer Luchavez, Mary Lorraine Mationg, Caroline Asiimwe, Audrey Albertini, Iveth J González, Michelle L Gatton, David Bell

**Affiliations:** 1 Foundation for Innovative New Diagnostics, Lumumba Avenue, Kampala, Uganda; 2 Department of Medical Microbiology and Parasitology, College of Medicine, University of Lagos, Idi-Araba, Lagos, Nigeria; 3 Research Institute for Tropical Medicine, Filinvest Compound, Alabang, Muntinlupa, Philippines; 4 Foundation for Innovative New Diagnostics, Avenue de Budé, Geneva, Switzerland; 5 Queensland Institute of Medical Research, Queensland, Australia; 6 Global Malaria Programme, World Health Organization, Geneva, Switzerland

## Abstract

**Background:**

Malaria rapid diagnostic tests (RDTs) are increasingly used by remote health personnel with minimal training in laboratory techniques. RDTs must, therefore, be as simple, safe and reliable as possible. Transfer of blood from the patient to the RDT is critical to safety and accuracy, and poses a significant challenge to many users. Blood transfer devices were evaluated for accuracy and precision of volume transferred, safety and ease of use, to identify the most appropriate devices for use with RDTs in routine clinical care.

**Methods:**

Five devices, a loop, straw-pipette, calibrated pipette, glass capillary tube, and a new inverted cup device, were evaluated in Nigeria, the Philippines and Uganda. The 227 participating health workers used each device to transfer blood from a simulated finger-prick site to filter paper. For each transfer, the number of attempts required to collect and deposit blood and any spilling of blood during transfer were recorded. Perceptions of ease of use and safety of each device were recorded for each participant. Blood volume transferred was calculated from the area of blood spots deposited on filter paper.

**Results:**

The overall mean volumes transferred by devices differed significantly from the target volume of 5 microliters (p < 0.001). The inverted cup (4.6 microliters) most closely approximated the target volume. The glass capillary was excluded from volume analysis as the estimation method used is not compatible with this device. The calibrated pipette accounted for the largest proportion of blood exposures (23/225, 10%); exposures ranged from 2% to 6% for the other four devices. The inverted cup was considered easiest to use in blood collection (206/226, 91%); the straw-pipette and calibrated pipette were rated lowest (143/225 [64%] and 135/225 [60%] respectively). Overall, the inverted cup was the most preferred device (72%, 163/227), followed by the loop (61%, 138/227).

**Conclusions:**

The performance of blood transfer devices varied in this evaluation of accuracy, blood safety, ease of use, and user preference. The inverted cup design achieved the highest overall performance, while the loop also performed well. These findings have relevance for any point-of-care diagnostics that require blood sampling.

## Background

Malaria rapid diagnostic tests (RDTs) are recommended for use in areas where good-quality microscopy is not available, including peripheral health centers and community-based case management programmes[1,2]. RDTs are, therefore, increasingly used by personnel with minimal training in laboratory techniques. To maintain test accuracy and utility in such settings the tests must be as simple, safe and reliable as possible. Previous studies have demonstrated that health workers with minimal formal training can satisfactorily perform and interpret RDTs, even with earlier and more complicated test formats[3-6]. However, reports and anecdotal observation have repeatedly indicated that blood transfer is an aspect of RDT use that poses a significant challenge to many users[7-12].

Most commercially available RDT kits are packaged with individual-use disposable blood transfer devices intended to collect, transfer and deposit a fixed volume of blood (typically 5 or 10 microliters) from a finger-prick to the absorbent pad within a well on the RDT cassette. Available transfer devices include loops, straw-pipettes, squeezable calibrated pipettes and capillary tubes. Blood transfer devices should fulfill a number of basic requirements: first, they should transfer the correct volume of blood (avoiding inaccurate test results); second, the risk of exposure of the user to direct blood contact through design, poor technique or accident should be minimized; and third, they should be easy to manipulate[13].

As RDT cassette design is fairly standardized, the parameters governing the appropriateness of design of the transfer device should be common to various RDT kits. Identifying the best design should therefore improve the overall diagnostic accuracy and safety of all RDT cassettes. Five devices were evaluated: four were typical examples of the main types of devices currently provided with commercially-available malaria RDTs, while the fifth was developed specifically for the study, based on an earlier promising design that had not been deployed with commercially-available tests. The study reported here evaluated blood transfer devices in terms of accuracy and consistency of blood volume transferred, blood safety, and ease of use in the hands of health workers, to identify the most appropriate blood transfer devices for use with malaria RDTs in routine clinical care.

## Methods

### Study participants

The study was conducted between August and November 2009 among 227 health workers in Nigeria, the Philippines and Uganda, drawn from staff of front-line health centers and community-based malaria programmes. Staff members with a patient care role were invited to participate irrespective of previous experience with RDTs, but information on previous experience with RDTs and blood transfer was recorded. Participants were unaware of the provenance of the devices evaluated, in order to avoid biasing the results. All participants provided written informed consent. The study protocol was approved by institutional review boards in each participating country.

### Devices evaluated

**Figure 1:**
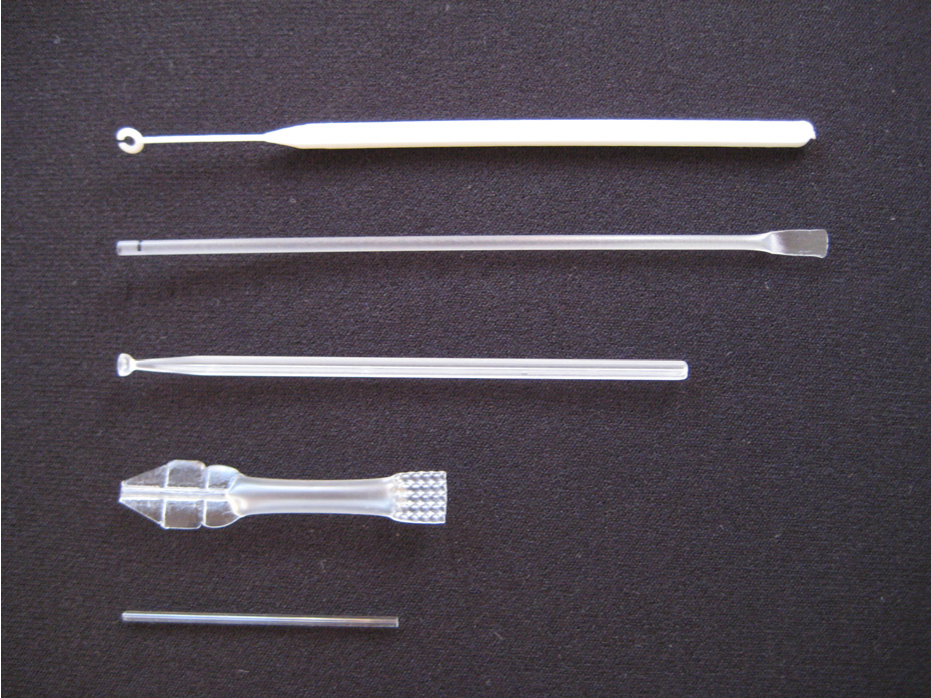
**Photograph of blood transfer devices evaluated**. From top to bottom, the loop, straw-pipette, inverted cup, calibrated pipette, and glass capillary.

The five blood transfer devices evaluated were the loop (hard plastic), straw-pipette (soft plastic), inverted cup (hard plastic), calibrated pipette (soft plastic), and capillary tube (glass), as shown in Figure 1. All except the inverted cup are currently packaged with various brands of commercially available RDTs that require a 5 microliter (μL) blood sample, while the inverted cup was manufactured for the study to a 5 μL design. The inverted cup consists of a concave "cup" 3 mm in diameter by 0.8 mm in depth, attached to a handle 8 cm long (Figure 1). The inverted cup device used in this study is made of polymethyl methacrylate. When applied to a sample of fresh or anticoagulated whole blood, the cup collects and retains blood by capillary action.

### Observed use of blood transfer devices

Prior to the evaluation, study staff demonstrated the correct use of each device to participating health workers, and allowed time for questions. Each participant then used each device under the observation of study staff, who recorded information on ease of use (blood collection, transfer and deposit) and safety (unintentional release of blood and blood exposure) using a checklist. A single sample of fresh venous blood in EDTA, pre-screened for infectious agents, was provided each day. To simulate collection and transfer of finger-prick blood whilst ensuring standardization, study staff pipetted 8-10 μL of venous blood onto a gloved finger. After five practice transfers with each device, each participant was observed using three devices of each type to transfer blood from the gloved finger to absorbent filter paper (Whatman grade 3) to simulate the absorbent pad of an RDT. Transfer devices were presented to each health worker in a computer-generated randomized order.

For each transfer attempt, the observer recorded whether a participant required more than one attempt to collect the desired amount of blood; whether blood was released unintentionally from the device at any time before reaching the target; whether the health worker required more than one attempt to deposit all the blood in the target area; and whether blood touched the health worker's gloves, skin or clothing at any time.

### Participant perceptions of utility

After each health worker had been observed transferring blood three times with all five devices, s/he was interviewed using a standard questionnaire regarding ease of collection, transfer and deposition of blood, and the perceived risk of blood exposure. Each health worker ranked the devices in order of preference and stated reasons for her/his choices.

### Measurement of transfer volume

The blood spots were dried and scanned, the area of each was measured using LineScale Plug-In version 1.80 (LineType Software Inc) [14] and the corresponding volume estimated using an area-to-volume coefficient generated each day from reference spots (below).

This method of volume estimation was used for four of the devices evaluated (loop, straw-pipette, calibrated pipette and inverted cup). However, the filter paper used in this assessment does not consistently draw blood from the glass capillary, and consequently a variable amount of blood remains in the capillary and the resulting blood spots do not accurately reflect its transfer volume; this phenomenon was confirmed with additional laboratory work at the Research Institute for Tropical Medicine (RITM) in the Philippines. Briefly, the full capacity of the glass capillary device was measured using a micropipettor and found to be 10 μL. Glass capillary devices were filled with EDTA-preserved blood and held steadily to RDT pads (n = 50) or filter paper (n = 50) for 10 seconds. The height of the blood column in each device was measured before and after the 10-second deposition time, and the difference was used to calculate the volume deposited onto each of the two surfaces. From a full starting volume of 10 μL, the mean volume transferred to the RDT pad was 6.59 μL, while the mean transferred to filter paper 2.23 μL, indicating a significant difference in the capacity of the two surfaces to wick blood out of the capillary tube. Therefore, the glass capillary results were disregarded from the analysis of blood volume transferred.

### Generation of reference blood spots and estimation of volumes transferred

Each day fresh venous blood was preserved in EDTA and study staff used a micropipette to make 20 reference blood spots of 5 μL each on filter paper. The area of each reference spot was measured with the LineScale software as above, and the mean was used as an area-to-volume coefficient for all spots produced that day from the same blood sample, ensuring that variations in haematocrit or other characteristics of the blood sample that could affect absorbance on filter paper did not bias results.

### Data management and statistical analysis

Data were entered into a central database using Epi-Info version 6.04 (Centers for Disease Control and Prevention, Atlanta, GA) and Microsoft Office Excel 2007, and analysed with Stata version 9 (Stata Corporation, College Station, TX). Proportions for each endpoint were calculated independently for each site and for all sites combined. Repeated measures ANOVA was used to test for differences in mean blood volume transferred between and within individuals. Mean volumes transferred were compared to the goal volume of 5 μL using a one-sample t-test. These analyses were performed in SAS version 9 (SAS Institute Inc.).

## Results

### Health worker participants

All participants in the Philippines were voluntary community health workers ("barangay health workers"); those in Nigeria included community health workers and facility-based health workers; most Ugandan participants were health workers employed at peripheral government health centers while the remainder were volunteer community health workers (Table 1). A majority of participants in Uganda (65/74, 88%) had previous experience with at least one of the devices, usually the loop, while prior experience with any blood transfer device was less common among participants in Nigeria and the Philippines (Table 1).

**Table 1:** Baseline characteristics of participating health workers

	Nigeria	Philippines	Uganda	Combined
Total participants	78	75	74	227

Dates	Oct 2009	Oct - Nov 2009	Aug, Nov 2009	---

Health worker category	78 (100%) facility-based clinical staff	75 (100%) village health workers	59 (80%) facility-based clinical staff; 15 (20%) voluntary/community workers	---

Had used any blood transfer device before evaluation date	13 (17%)	8 (11%)	65 (88%)	86 (39%)

Used loop before	0	1 (1%)	65 (88%)	66 (29%)

Used straw-pipette before	0	1 (1%)	21 (28%)	22 (10%)

Used glass capillary before	10 (13%)	3 (4%)	8 (11%)	21 (9%)

Used calibrated pipette before	1 (1%)	3 (4%)	33 (45%)	37 (16%)

Used inverted cup before	2 (3%)	0	0	2 (1%)

Used other device before	0	0	5 (7%)	5 (2%)

### Accuracy and precision of blood volume transferred

**Figure 2:**
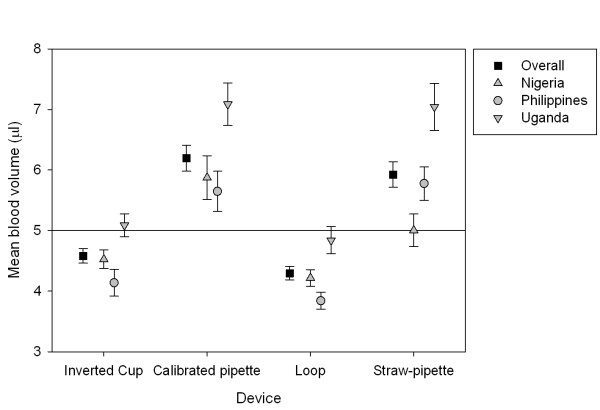
**Blood volumes transferred by each device**. Squares, triangles and circles represent total and site-specific mean average volumes. Whiskers represent 95% confidence intervals. NB: The glass capillary is excluded from this analysis because the filter paper surface used in this study does not consistently draw blood from the capillary device, and consequently the resulting blood spots do not accurately reflect its transfer volume.

Blood volumes transferred using each device are presented in Table 2 and Figure 2. Values shown in the table are those obtained when the transfers made with each device by each health worker (three transfers per health worker per device) are aggregated (i.e. the unit of analysis is each health worker); there were no significant "within health worker" effects in the repeated measures ANOVA (p > 0.3). The glass capillary is disregarded from this analysis (see Methods). Of the remaining four devices, the average volume deposited by the inverted cup (4.6 μL) most closely approximated the desired volume of 5 μL; the loop delivered slightly less (mean 4.3 μL), while the straw-pipette and calibrated pipette delivered more (mean 5.9 μL and 6.2 μL, respectively). There was significant variation in mean volumes deposited by each device among the Ugandan, Philippine and Nigerian sites. All mean blood volumes deposited differed significantly from the desired volume of 5 μL (p < 0.001) with the exception of the inverted cup in Uganda, loop in Uganda and straw-pipette in Nigeria (p > 0.1).

**Table 2:** Accuracy and precision of blood volumes transferred by health workers with each device

	Nigeria 78 users		Philippines 75 users		Uganda 74 users		All Sites Combined 227 users	
	
	**Volume in μL**^**a**^ mean (std dev), range	**p-value**^**c**^	**Volume in μL**^**a**^ mean (std dev), range	**p-value**^**c**^	**Volume in μL**^**a**^ mean (std dev), range	**p-value**^**c**^	**Volume in μL**^**a**^ mean (std dev), range	**p-value**^**c**^
LOOP	**4.219** (0.603) 2.803 - 5.804	<0.0001	**3.842** (0.607) 2.597 - 5.312	<0.0001	**4.841** (0.968) 2.845 - 9.306	0.1611	**4.297** (0.846) 2.597 - 9.306	<0.0001

STRAW-PIPETTE	**5.004** (1.191) 1.796 - 7.602	0.9768	**5.778** (1.199) 2.922 - 9.551	<0.0001	**7.042** (1.671) 2.038 - 9.469	<0.0001	**5.924** (1.601) 1.796 - 9.551	<0.0001

GLASS CAPILLARY^b^	**---**	---	**---**	---	**---**	---	**---**	---

CALIBRATED PIPETTE	**5.875** (1.597) 2.259 - 10.287	<0.0001	**5.649** (1.437) 2.681 - 10.406	0.0002	**7.086** (1.507) 3.444 - 11.855	<0.0001	**6.195** (1.635) 2.259 - 11.855	<0.0001

INVERTED CUP	**4.525** (0.683) 2.371 - 5.885	<0.0001	**4.139** (0.958) 2.874 - 7.202	<0.0001	**5.088** (0.824) 3.499 - 7.382	0.3633	**4.581** (0.910) 2.371 - 7.382	<0.0001

^a ^Estimated from the areas of individual blood spots made by transferring blood to absorbent filter paper.

^b ^The glass capillary is excluded from this analysis because the filter paper surface used in this study does not consistently draw blood from the capillary device, and consequently the resulting blood spots do not accurately reflect its transfer volume.

^**c **^Comparison of mean volume with goal volume of 5 microliters (μL).

### Ease of use and blood safety

Overall, more than one-third of the health workers required at least one repeat attempt to collect blood with the loop (86/226, 38%), the straw-pipette (97/224, 43%) and the calibrated pipette (91/226, 40%), while the other two devices required fewer repeat attempts (25/225 [11%], and 18/221 [8%] for the glass capillary and inverted cup respectively, Table 3). There were few instances of inadvertent release of blood during transfer, with the highest number occurring with the calibrated pipette (9/226 [4%], all in Nigeria). The glass capillary required the highest number of repeated attempts at deposition or release of blood (60%), though this may have been due in part to the use of filter paper rather than the more absorbent pad used in some RDTs (see Methods). The use of the calibrated pipette accounted for the largest number of blood exposures (23/225, 10%); exposures ranged from 2% to 6% for the other four devices (Table 3). Observed difficulty in device manipulation varied between sites, with the Ugandan participants recorded as having the least difficulties overall.

**Table 3:** Observation of health workers' use of devices for blood collection, transfer and deposition

	Nigeria **n = 78**^**a**^	Philippines **n = 75**^**a**^	Uganda **n = 74**^**a**^	Combined **n = 227**^**a**^
	
	Number (%) observed "Yes" for any of the health worker's 3 transfers per device			
**≥ 1 failed attempt to collect blood**				
LOOP	21/77 (27%)	49/74 (66%)	16 (22%)	86/225 (38%)

STRAW-PIPETTE	43/77 (56%)	32/73 (44%)	22 (30%)	97/224 (43%)

GLASS CAPILLARY	13/77 (17%)	8/74 (11%)	4 (5%)	25/225 (11%)

CALIBRATED PIPETTE	54 (69%)	23/74 (31%)	14 (19%)	91/226 (40%)

INVERTED CUP	3/75 (4%)	14/72 (19%)	1 (1%)	18/221 (8%)

**Blood released unintentionally during transfer**				
LOOP	0/76	1/73 (1%)	1 (1%)	2/223 (1%)

STRAW-PIPETTE	1/76 (1%)	0/73	1 (1%)	2/223 (1%)

GLASS CAPILLARY	2/76 (3%)	1/73 (1%)	0	3/223 (1%)

CALIBRATED PIPETTE	9 (12%)	0/74	0	9/226 (4%)

INVERTED CUP	0/75	0/72	1 (1%)	1/221 (0.5%)

**≥ 1 failed attempt to deposit blood**				
LOOP	7/75 (9%)	17/73 (23%)	4 (5%)	28/222 (13%)

STRAW-PIPETTE	24/77 (31%)	12/73 (16%)	11 (15%)	47/224 (21%)

GLASS CAPILLARY^b^	78 (100%)	35/73 (48%)	23 (31%)	136/225 (60%)

CALIBRATED PIPETTE	16/77 (21%)	11/74 (15%)	9/74 (12%)	36/225 (16%)

INVERTED CUP	13/72 (18%)	28/71 (39%)	8 (11%)	49/217 (23%)

**Health worker exposed to blood**				
LOOP	12/77 (16%)	1/72 (1%)	0	13/223 (6%)

STRAW-PIPETTE	7/76 (9%)	3/70 (4%)	2/73 (3%)	12/219 (5%)

GLASS CAPILLARY	3/73 (4%)	5/72 (7%)	1 (1%)	9/219 (4%)

CALIBRATED PIPETTE	18/77 (23%)	3/74 (4%)	2 (3%)	23/225 (10%)

INVERTED CUP	4/74 (5%)	0/72	0	4/220 (2%)

^a ^Where alternative "n" specified, the balance had blank data in the case record forms.

^b ^The glass capillary's results for blood deposition were negatively affected by the fact that the filter paper surface used in this study does not consistently draw blood from the capillary device, and therefore it was more difficult for study participants to deposit blood using this device.

Opinions of health workers on ease of use, risk of blood exposure, and appropriateness of each device are summarized in Table 4. The inverted cup was considered easiest to use in blood collection (206/226, 91%); while the straw-pipette and calibrated pipette were ranked lowest (143/225 [64%] and 135/225 [60%] respectively). All devices except for the glass capillary ranked highly in ease of deposition, with the inverted cup and loop rated easiest by 93% (211 and 210 out of 226, respectively). A relatively low proportion of participants perceived a risk of blood exposure with the inverted cup (12/224, 5%); those perceiving risk for the other devices ranged from 14% to 22% (Table 4). The inverted cup was considered "appropriate for health workers to use in patient care" by a large majority of participants (210/225, 93%), followed by the loop with 79% (178/226) (Table 4). More detailed explanations given by health workers for their responses are presented on-line in Additional file 1, Table S1.

**Table 4:** Participant questionnaire: ease of use, risk and appropriateness of each device^a^

	Nigeria **n = 78**^**b**^	Philippines **n = 75**^**b**^	Uganda **n = 73**^**bc**^	Combined **n = 226**^**b**^
	
	Number and (%) of health workers answering "Yes"			
**Easy to collect (pick up) blood**				

LOOP	77 (99%)	30 (40%)	67 (92%)	174 (77%)

STRAW-PIPETTE	42 (54%)	61/74 (82%)	40 (55%)	143/225 (64%)

GLASS CAPILLARY	72 (92%)	63/74 (85%)	50 (68%)	185/225 (82%)

CALIBRATED PIPETTE	42 (54%)	61/74 (82%)	32 (44%)	135/225 (60%)

INVERTED CUP	78 (100%)	57 (76%)	71 (97%)	206 (91%)

**Easy to release (deposit) blood**				

LOOP	78 (100%)	63 (84%)	69 (95%)	210 (93%)

STRAW-PIPETTE	61 (78%)	68/74 (92%)	61 (84%)	190/225 (84%)

GLASS CAPILLARY^d^	9 (12%)	28/74 (38%)	34 (47%)	71/225 (32%)

CALIBRATED PIPETTE	61 (78%)	68/74 (92%)	66 (90%)	195/225 (87%)

INVERTED CUP	78 (100%)	65 (87%)	68 (93%)	211 (93%)

**Risk of blood exposure**				

LOOP	1/76 (1%)	22/74 (30%)	9 (12%)	32/223 (14%)

STRAW-PIPETTE	5 (6%)	7/73 (10%)	19 (26%)	31/224 (14%)

GLASS CAPILLARY	21 (27%)	11/71 (15%)	17 (23%)	49/222 (22%)

CALIBRATED PIPETTE	14 (18%)	5/73 (7%)	17 (23%)	36/224 (16%)

INVERTED CUP	0/77 (0%)	10/74 (14%)	2 (3%)	12/224 (5%)

**Appropriate for health workers to use in patient care**				

LOOP	76 (97%)	37 (49%)	65 (89%)	178 (79%)

STRAW-PIPETTE	47 (60%)	63/73 (86%)	39 (53%)	149/224 (67%)

GLASS CAPILLARY^d^	22 (28%)	48/71 (68%)	36 (49%)	106/222 (48%)

CALIBRATED PIPETTE	37 (47%)	63/73 (86%)	36 (49%)	136/224 (61%)

INVERTED CUP	78 (100%)	61/74 (82%)	71 (97%)	210/225 (93%)

^a ^See on-line Supplementary Table for more detailed qualitative data on health workers' reasons for these answers.

^b ^Where alternative "n" specified, the balance had blank data in the case record forms.

^c ^One participant did not answer questions in this section.

^d ^The glass capillary's results for blood release were negatively affected by the fact that the filter paper surface used in this study does not consistently draw blood from the capillary device, and therefore it was more difficult for study participants to release or deposit blood using this device.

### Health workers' preferences

Ranking of devices by health workers as 'best' and 'worst' for use with malaria RDTs provided similar results (Table 5). The inverted cup was most preferred (72%, 163/227), followed by the loop (61%, 138/227). The glass capillary was least preferred (40%, 90/227). In general, the inverted cup and loop were preferred on the basis of ease and speed of blood collection and deposition and a perceived low risk of blood exposure (Table 5).

**Table 5:** Preferences of health workers among devices evaluated, and reasons given

Device and number (%) of health workers ranking it *most or second *preferred	Representative reasons given for listing device as preferred
**LOOP **138 (61%)	"Doesn't waste time, easy to collect, transfer, release and manipulate." "Easy to collect, transfer and deposit with minimal blood exposure risk." "Doesn't require measurement, a single touch collects and deposits the blood, no squeezing is required." "I've used it before, and it's simple to use; also time-saving." "Easily picks the sample; doesn't have sharp edges which could cause fear to the patient."

**STRAW-PIPETTE **53 (23%)	"Pick up blood fast and easy to deposit, no worries that it will spill." "Easy to use and not breakable." "Accurate, fairly safe, easy to use, easy to release blood." "Has a mark where sample should end, you apply pressure so you have control of it."

**GLASS CAPILLARY **44 (19%)	"Easy to collect and easy to transfer, blood not scattered." "It draws and releases blood automatically." "Might waste some time, but doesn't need any manipulation, so very easy."

**CALIBRATED PIPETTE **56 (25%)	"Adequate volume, no blood spillage, easy to collect and to deposit." "Fast, no spills and soft to use, fast in picking up." "Person is aware of right amount of blood to obtain because it's marked." "Picks up quickly, no exposure."

**INVERTED CUP **163 (72%)	"It is the easiest and it saves time." "Easy to collect, transfer, deposit with minimal blood exposure risk." "Faster, very easy to use, no spillage, user friendly and accurate." "Easy to pick the blood, even depositing it is easy; because it's enclosed in the cup, no risk of getting exposed to the blood." "Easy to collect, release and doesn't need any technician training." "Blood remains accommodated in the cup and doesn't splash even on shaking; it's safe." "No technique is required; no measurements needed."

**Device **and number (%) of health workers ranking it *least *preferred	**Representative reasons given for listing device as *least *preferred**

**LOOP **41 (18%)	"Difficult in collecting and transferring, might spill blood during transfer." "Tendency to be spilled and only small amount of blood is obtained." "It's challenging to collect the blood from finger."

**STRAW-PIPETTE **28 (12%)	"Difficulty in pressure manipulation to collect blood." "It takes time to suck the blood since it requires much squeezing." "You may press, as you release, too much blood goes in; difficult to measure blood to the mark."

**GLASS CAPILLARY **90 (40%)	"Small and difficult to hold, easily broken." "It's so automatic that one can't order it to speed up so it does take time." "Makes picking blood difficult with children because of time duration, and it may break because of brittleness and prick me or my patient." "So tiny someone with sight problems can find it difficult; you must be careful, it does not give you speed, so you may find your patients lining up." "It may break injuring the patient or the health worker; it takes time; it may scare children thinking that they may be pricked for the second time."

**CALIBRATED PIPETTE **63 (28%)	"Only appropriate for people with good sight and steady hands; may not be appropriate for all health workers." "It has many risks, it can splash blood, it can release before you reach where you want to put blood." "There's some difficulty, you need some technique to get the blood to the mark, the right amount; and if you have a kid who is fidgeting, you may lose the pressure and the blood goes." "It has been hard for me to measure here [to the mark]; when I try to draw to the mark, it goes past; when I try to make the blood go down to the mark, it goes all out fast; so it takes time."

**INVERTED CUP **5 (2%)	"Had difficult in collecting blood, small amount goes in." "Retained the blood samples, I had to bend it if it refused to release blood."

## Discussion

As reliability, blood safety, and ease of use of RDT test kits will be fundamental to the success of large-scale implementation of parasite-based diagnosis, optimizing blood transfer devices is important. The five blood transfer devices assessed here, four of which are representative of the devices commonly used with commercially-available malaria RDTs, and one of a more novel design, exhibited a wide range of performance characteristics.

In this study, the inverted cup device provided the best overall performance, and was considered the most appropriate choice for use with RDTs by the majority of participating health workers. The loop also performed well, with similar accuracy and precision of blood volumes transferred but slightly lower scores for other characteristics. The other devices evaluated, including a squeezable straw-pipette, squeezable plastic calibrated pipette, and glass capillary, provided lower accuracy and precision in blood transfer, higher risk of blood exposure, and lower scores on ease-of-use assessment and user preference. The main advantages of the cup design appear to result from the ease of uptake or collection of blood (achieved by contact with the blood drop surface) and presentation of a broad surface area of retained blood for release onto the absorbent surface. In contrast, the loop presents a narrow contact surface unless tilted sideways (which some participants noted is more challenging when depositing blood into the well of an RDT rather than onto the flat surface of the filter paper used in this evaluation). The straw-pipette and calibrated pipette also required further manipulation to ensure contact between the blood and the absorbent surface.

The need for improved blood transfer methods for use with RDTs has been identified in previous reports[7-12]. The only previously published direct comparison of malaria RDT blood transfer devices, by Luchavez *et al *[13], assessed blood volumes and ease of use of devices available at the time and assessed the importance of variation in blood volume to RDT accuracy. Although excess blood did not affect accuracy, it was noted that blood staining of the RDT test strip may obscure test results[13]. Inadequate blood volume is likely to become critical at low parasite densities, when low volume reduces RDT sensitivity. In the assessment by Luchavez *et al *sensitivity was considerably reduced at parasite densities of 200 parasites/μL with volumes less than 3 μL for RDTs designed for 5 μL of blood[13]. In the present study, for all devices at all sites, both the mean volume transferred and the mean minus one standard deviation fell above 3 μL (Table 2), although the range fell below 3 μL in some cases. The reason for differences among the three sites in volumes transferred is not clear, as identical methods and materials were used at all sites; it may be speculated that differences in blood samples and ambient conditions, or possibly inadvertent differences in technique, may have led to variation in how the blood was absorbed by the filter paper.

A limitation of this evaluation is the somewhat artificial condition created by using absorbent filter paper, rather than RDT pads, for blood deposition. This method allowed us to estimate the blood volumes transferred by each device in the hands of health workers, but provided unreliable results for transfer volume with the glass capillary due to the observed difference in the absorbent qualities of filter paper compared to an RDT pad. The same phenomenon, and the resultant difficulty in depositing blood onto the filter paper from a glass capillary, may also have contributed to health workers' less favorable perception of this device, although many participants also commented on unrelated features. Another artificial condition created for this study was the placement of a blood drop on a gloved finger, rather than pricking a patient's finger, to obtain a source of blood for each transfer. The devices tested may perform slightly differently when collecting blood from skin rather than the latex surface.

The current study was specifically aimed at assessing the suitability of the devices for use by front-line health workers with limited laboratory training and limited facilities. Some workers may also have lesser capacity for fine manipulation. Clearly, specific training may overcome some of the apparent deficiencies illustrated here. For example, the capillary tube and calibrated pipette may perform better in laboratories with technicians accustomed to manipulating such devices. The results of this study show significant variation between study sites; differences in prior training or experience using certain types of devices are likely explanations for these variations. For example, Ugandan health workers who have considerable experience with RDT use made, in general, fewer observed errors with all devices than did less-experienced participants in the other two countries (Table 3), although patterns across the sites were not always consistent and differences were not often large. Where health workers are already trained and accustomed to a particular device design, the process of change may have a detrimental impact on overall performance, which would outweigh the potential advantages of a new design. However, when considering large-scale implementation of RDTs in remote areas, essential to fulfill the new global WHO policy of parasite-based diagnosis prior to anti-malarial treatment,[2] performance, safety and ease of use in the hands of front-line health workers will be critical to success.

As RDT cassette design is essentially standard, blood transfer devices should be an exchangeable accessory. Careful selection of the blood transfer device should be a part of overall RDT kit selection. Flexibility in matching the most appropriate RDT (in terms of detection threshold, species detection, stability and cost) to the most appropriate transfer device for the field of intended use could improve the outcome of RDTs' introduction into malaria programmes.

In addition to malaria RDTs, point-of-care tests based on sampling of small quantities of blood or serum are currently used at peripheral health care levels for a number of diseases (e.g. dengue, syphilis), and will be of increasing importance as new tests are developed for other diseases. The results of this study are likely to be applicable to these tests, though they should be confirmed on different sample types and with different cadres of health or laboratory worker. It seems illogical to use a wide variety of device designs aimed at achieving essentially the same task, when required transfer volumes are similar, and accuracy, blood safety and ease of use are universally important issues. Establishing quality and consistency in the design of point-of-care diagnostic kits for different diseases will improve both diagnostic performance on the front lines of the health care system and ease of training and implementation.

## Conclusions

The performance of blood transfer devices varied in this evaluation, with the inverted cup design achieving the highest overall performance in terms of accuracy and precision of blood volume transferred, blood safety, ease of use, and user preference, while the loop also performed well in most respects. The relative appropriateness of the different device designs may vary in other settings and in the hands of personnel with different levels of training. These findings have relevance for any point-of-care diagnostics that require blood sampling, and the variability in device performance demonstrated here highlights the need to devote attention to this issue when implementing RDT-based diagnostic programmes.

## Competing interests

The authors declare that they have no competing interests.

## Authors' contributions

HH designed the study, contributed to development of the inverted cup device, coordinated field work in Uganda, analysed and interpreted the data, and drafted the manuscript. WO contributed to study design and coordinated field work in Nigeria. JL and MLM contributed to study design, coordinated field work in the Philippines, and conducted additional laboratory assessments at RITM. CA contributed to study design and participated in critical revision of the manuscript. AA and IG coordinated development of the inverted cup device, contributed to study design and participated in critical revision of the manuscript. MLG performed the statistical analysis for blood volumes transferred and participated in critical revision of the manuscript. DB conceived the study, contributed to development of the inverted cup device, contributed to the study design, and helped to draft and revise the manuscript. All authors read and approved the final manuscript.

## Supplementary Material

Additional file 1 **Table S1. Explanations given by participants for perceptions of ease of use, risk and appropriateness as summarized in Table 4 in the full manuscript**. Click here for file
