# Supplementary material for: Blood transfer devices for malaria rapid diagnostic tests: evaluation of accuracy, safety and ease of use
Source: Malar J. 2011 Feb 8;10:30. doi: 10.1186/1475-2875-10-30 (PMC3041722; doi:10.1186/1475-2875-10-30)
Supplement: Additional file 1 — Table S1. Explanations given by participants for perceptions of ease of use, risk and appropriateness as summarized in Table 4 in the full manuscript. [file 1475-2875-10-30-S1.DOC]

**SUPPLEMENTARY DATA: BLOOD TRANSFER DEVICE EVALUATION**

**Supplementary on-line Table 6. Explanations given by participants for perceptions of ease of use, risk and appropriateness as summarized in Table 4 in full manuscript**

|  | **Representative explanations given for answers in Table 4** |
| --- | --- |
| **LOOP, collection: Yes, easy**  Nigeria | does not require energy / effort  just a single touch needed / easy and requires just a touch  it saves time / does not waste time / it is faster  easy to operate / easy to manipulate  it is not stressful / pressure is not needed |
| Philippines | after touching, blood immediately goes with it / just a touch with the blood and it goes immediately with the device / after placing, blood goes with it automatically  if placed exactly in blood, blood easily adheres to it  collects blood fast  (13 blank) |
| Uganda | on touching the blood gets in easily / it needs only a touch / only requires a single touch to collect blood / easy to use, it was a matter of touching the blood / loop picks up blood faster as soon as it touches the drop  it required touching the blood and the hole filled by itself / matter of putting it and appropriate blood will come automatically  it’s long enough, and immediately you put it in blood it draws the blood, does not waste time  once you put it in blood, it has the capacity to pick the appropriate amount of blood easily and very fast  the moment it dips in, it scoops up the blood, so it was easy for me  appropriate angle for it to touch blood  does not require pressing to draw blood / no pressure needed to draw blood, draws blood by tip  I have used it before, you just put [it on blood] and it sucks into the circle blood which is enough  I just tap, doesn’t need any manipulation  you just “pip,” no need to force it, but it needs enough blood on the finger  you just put there – ah! – it picks  it was quicker thus time saving |
| **LOOP, collection: No, not easy**  Nigeria | because it does not collect blood easily |
| Philippines | blood might drop while still in the patient’s fingers  blood does not go with the device immediately  blood needs to be in a lump for easy collection, if not lumped it is difficult to collect  only small amount of blood goes in / difficult to collect because only small amount of blood goes with it / only small amount of blood is obtained  hole is not uniform and blood does not stick immediately  blood does not go in immediately / blood takes time going in  (5 blank) |
| Uganda | it requires a large volume or drop of blood  you need some tact in using loop, unless the blood is much  easy with an adult who is fixed, but not with a kid who is fighting, or where blood is not enough  the loop may not be filled with blood |
| **LOOP, deposit: Yes, easy**  Nigeria | easy to manipulate while depositing blood / easy to use while depositing blood  not stressful / not difficult  it only requires a touch / just single touch also / once you touch the paper, the paper absorbs it  does not waste time / just little touch is needed to release blood  no need to press  no spillage if done easily |
| Philippines | after pressing, blood immediately drops  after touching the filter paper, blood is immediately placed  releases blood fast  (25 blank) |
| Uganda | it releases blood by a single touch / it releases all the blood on a single touch / because it just required touching the filter paper and the blood went/absorbed / once you put it on surface, the blood comes out  needs no technicality; just a touch on the paper and blood comes out / you just press it on the part, don't need any pressure or mechanism  release blood without pressure applied  you simply place, and blood drains itself / you put it on the target, the blood gets out by itself  it takes little time since the blood spreads faster / doesn't take a lot of time / it took a short time / it releases blood very fast  you just have to tap with hole facing the surface  bending it to enable the blood to be absorbed by the filter paper  didn't require any experience even a layman can use it |
| **LOOP, deposit: No, not easy**  Nigeria | (no “no” answers) |
| Philippines | needs to be folded before placing blood  only a small amount of blood is obtained *or* transferred  not all blood is obtained because it sticks in the hole / takes time to collect and deposit, there are some blood left in the device  (2 blank) |
| Uganda | tricky, blood might splash if loop bends as you press to release blood  I expected it to come from opening at tip but at times I had to tilt it a bit / it required bending it in order to release |
| **LOOP: Yes, risk of exposure**  Nigeria | during blood collection |
| Philippines | blood may drop while transferring  blood is only outside, nothing to hold it / might spill because it is open / it has holes / has a hole  if the hands of the BHW are shaky it can spill on him  if patient is fidgety blood can spill on him  it's open and there's a possibility that it will be spilled by children  not safe because even with slight movement, blood splatters  (4 blank) |
| Uganda | there is a risk of blood dropping off  carries an external drop which can touch your body/clothes or even splash into eye  blood is exposed, outside, not inside tube, so it can easily drop on us / blood is basically outside and can be spilt out easily  blood easily spills if one panics or shakes  maybe if patient is a child who is shaking, the health worker could get splashed |
| **LOOP: No, not appropriate**  Nigeria | because it is open, there is a tendency for blood exposure  was not taking enough blood |
| Philippines | blood may drip out / blood does not hold / might spill because it is open / not safe, blood can fall  slow in obtaining blood / takes time in collecting blood  not easy to collect blood / difficult to collect blood / difficult to collect if blood is spread  in terms of collection it is not appropriate for young patients  (6 blank) |
| Uganda | because it is not easy to fill the loop / just because the hole may not be filled with blood  because of fear of blood dropping off  removing blood takes a lot of time  tricky to release blood, and risk of splashing or exposure  does not favor all categories of patients because difficult with struggling children |
| **STRAW, collection: Yes, easy**  Nigeria | once you apply pressure, blood flows / with little pressure you get blood in / you only need to press for the blood to flow in  but with tendency to collect excess due to pressure manipulation / but you need to be careful with pressure manipulation  (1 blank) |
| Philippines | can absorb blood fast / blood enters fast / fast in obtaining blood  when pressed properly blood goes with the device easily / just pinch and blood immediately enters  just pinch and blood immediately enters, should have timing so that blood doesn’t enter too far into the device  easy to collect but should have control of hands / just needs control or too much blood will be obtained  soft and able to control in obtaining blood  (16 blank) |
| Uganda | with practice / was later easy to take the required amount of blood  has a label to guide the amount of blood required  it absorbs up to the mark and easy to manipulate pressure / easily picks, gentle sucking pressure easily measures to mark  it has a mark, there is even a pressure you put, it gets the amount you want, it’s controllable  it is easily pressed and the blood flows in it / needs little pressure to absorb blood / easy to apply pressure and then releasing to draw blood  just you press and the pressure sucks the blood from the fingerprick  easy but you have to observe the point while collecting |
| **STRAW, collection: No, not easy**  Nigeria | I was not taking enough blood because of difficulty in pressing  a lot of pressure must be applied / pressure needed is much  problem of manipulating pressure to suck blood / difficulty in pressing and releasing (pressure problem) / it is difficult because of the pressure it requires  pressure manipulation problem, tendency to take more than necessary blood  picks more than the required volume at times / it takes more than required amount of blood / too much blood can be collected  requires technical effort and there's need for good vision because of mark  because it wastes time  very difficult and needs training to manipulate it  the capillary is thin and difficulty in pressure manipulation |
| Philippines | difficult because pressing needs to have timing to obtain blood / blood has difficulty to be suctioned because it needs to go with timing  it is hard when it has something to press, the blood becomes messy  confused because difficult to press  it folds, difficult to collect  (1 blank) |
| Uganda | it requires a lot of squeezing  because of squeezing and timing the mark  needs pressing which is difficult (also tiny)  it was difficult to estimate the exact pressure required to suck blood / was difficult to estimate the desired pressure to obtain blood / I don’t know how much pressure to apply, too little doesn't reach mark and too much goes past / it was difficult to balance pressure to collect the desired volume  it is difficult to collect the right volume of blood to the mark / it's difficult to collect blood up to the mark / not easy to draw exactly at the mark  you have to be strict and be sure the blood does not go beyond this black mark  has so many techniques to take in mind, pressing, not pressing to patient’s finger  I would first press before entering blood drop, it required some technique and practice  you have 1st to press, release, and if you release so quickly, blood goes in much  the pressing and releasing process is confusing in blood collection  a bit long, in the process of pressing I found it difficult to point into blood  sometimes I'd exceed the mark that is put, and if you get too much blood you can get wrong results |
| **STRAW, deposit: Yes, easy**  Nigeria | through pressure blood is easily released / with small pressure it goes out / with small pressure it is easily released / just press to release blood  easy to manipulate  easy to use and is not stressful  (4 blank) |
| Philippines | easily released when pressed / when pressed blood released right away / after pressing, blood immediately drops / just pinch and blood will come out / one press and blood immediately drops  just needs to be synchronized  (23 blank) |
| Uganda | needed little pressure to drop blood / releases easily on squeezing  you just squeeze and it goes very fast / immediately you press, the blood comes out  it requires only slight squeezing / it just required pressure to release  just a matter of maintaining pressure and pressing harder  once blood already in, releasing was easy, I did not find any problem |
| **STRAW, deposit: No, not easy**  Nigeria | pressure manipulating problem / because of pressing and release  pressure must be exerted and full concentration  didn't dispose blood completely  (1 blank) |
| Philippines | difficult to pinch / difficult to pound out  blood doesn't come out no matter how much you press  it folds, needs to be twisted  (1 blank) |
| Uganda | it needs a lot of effort and concentration in squeezing  required much pressure in order to release blood  pressing didn't release blood well - spilt blood  required a lot of pressing to release (contrary to calibrated pipette)  walls retained blood, some would retain, also a question of how much pressure to apply  you might fail to release unless you exert pressure properly  if you release before reaching the site, blood can go up (in the straw) and you end up putting less blood on the site  it needs a technique, someone to explain, a bit of experience |
| **STRAW: Yes, risk of exposure**  Nigeria | spillage can occur during pressure application  pressure manipulation problem may cause spillage |
| Philippines | if blood is not obtained immediately, it is spilled  (5 blank) |
| Uganda | blood may easily drop when over-squeezed / the blood may drop off when squeezed  manipulating the pressing and releasing may make blood spill out easily  trembling resulting in splashing the sample / because I was quaking  some bubbles formed, putting her at risk of blood exposure  she felt that she could press/squeeze before reaching the filter paper / supposing you release before reaching the target, blood may come out / when you transfer sample from patient, you may squeeze and blood drops anywhere  it's long, as you transfer if you knock something it may even splash  as you press, straw might bend, and then it could splash / very flexible and staggers when in blood and splashes blood  you need to be gentle while releasing the blood, or it can splash  it is difficult to be used in children |
| **STRAW: No, not appropriate**  Nigeria | it will be difficult to health workers to get appropriate volume required  not easy to pick blood due to inability to manipulate pressure / difficulty in manipulating pressure  because of difficulties in sucking up and releasing, not user friendly / pressure manipulation is not user friendly  because of difficulty in sucking up and releasing / it doesn't suck or release blood easily  difficulty in application of pressure and there is risk of spillage  it requires supervision  it may waste time / takes time and requires patience  (4 blank) |
| Philippines | had difficulty using because when not in exact place the blood becomes messy  has difficulty, blood might dry  (7 blank) |
| Uganda | it is difficult to get blood to the mark and it consumes time / estimating to the mark is difficult to attain or achieve / estimating blood to the mark is difficult  it takes time to get blood / it will take long time  it can delay taking blood, because of passing mark when squeezing, unlike other devices which are immediate  just because of the health worker with a tendency of shaking  blood exposure risk too high especially when patients are many  technicality in drawing and releasing exposes people to blood  not easy to suck and release blood, can spill blood  may draw too much blood and/or fail to release, risk of blood exposure  this fidgeting with collection and release causes loss of confidence, even me I fear because not knowing pressure, and the patient also fears  because one has to repeat many times in order to get used to it  needs experience and care, must be very, very careful, with children, it can complicate  if a health worker has poor sight, they can't see the mark and may find it difficult  if you're not very particular, blood can go a little bit more than the mark  to find how much pressure to exert may be hard for some people  wastes time and exposes people to risk of real working environment |
| **GLASS CAPILLARY, collection: Yes, easy**  Nigeria | the blood flows easily / blood flows in easily  it is easy to operate and blood flows in easily / easy to manipulate and blood flows in easily  blood flows in easily though you need to bend it sideways  (9 blank) |
| Philippines | easily collects blood, precaution needed in collection since it is breakable  just slight touch with blood and it goes in immediately but needs care to not break it  just a slight touch and the blood goes immediately with the device / blood enters immediately / after placing in the blood, it enters immediately  fast / suctions blood fast  fast, no need to press, blood immediately enters  easy to collect blood, just put it at the center / just put near the blood, it absorbs immediately  no more pressing, blood goes in immediately / no need to press  easy because with right timing, it enters immediately, not messy and not unstable  (26 blank) |
| Uganda | it sucks blood by itself / it draws blood by itself / it draws blood to the full by itself / the blood was just drawn by capillary action / blood moved by itself by capillary action  the blood moved by itself and even stopped by itself / it sucks the blood and stops by itself / it's automatic, it picks, when satisfied, it stops  blood flows into it automatically / it automatically draws blood / it's automatic, needs no pressure / coz it's automatic  blood moves up the tube without any pressure applied / capillary glass draws blood by itself without any pressure  you don't need to put any pressure on it, like the others  it uses capillary attraction, don't need any pressure or mechanism  you just need to put it in between blood (not on skin), needs some technique  you just put + automatically comes, it even shows you how much, doesn’t require energy  the moment you put it on blood, the blood fills in adults; in children it may not be easy |
| **GLASS CAPILLARY, collection: No, not easy**  Nigeria | it takes a lot of time for blood to be collected / it is time wasting / it takes time  not fast enough and user friendly as compared with others |
| Philippines | easily broken / had difficulty because he was afraid to break it  difficult when only small amount of blood  difficult to collect and there are some blood spilled in the fingers  hole is small, blood takes time in entering |
| Uganda | it takes time to collect blood / it takes time to collect blood / takes time to draw uncontrolled / takes long to suck up blood  because of time it takes; might be OK for adult, but not children because they struggle / takes some time so could be difficult with children  on touching the skin of the patient it blocks thus doesn't collect blood  very tiny and difficult to see drawing blood  sometimes it sucks little blood, you don't have much control  if a real patient, without an adequate drop of blood, or with a child, it would be difficult to observe if the capillary is still absorbing  in order to get blood, you observe to see where blood starts going and when it stops, so it takes some time  when picking blood, he thought blood had stopped moving, yet it was still moving; it’s short  putting it on a patient, I would shake, because it's small  my worry is with young children who fight, because it must be held in one place to fill |
| **GLASS CAPILLARY, deposit: Yes, easy**  Nigeria | blood is deposited in a designated area  with time all the blood will be deposited  (1 blank) |
| Philippines | blood can be delivered right away  blood goes down by itself  blood easily comes out and not scattered  because it was controlled / even if slow but surely places blood  just a slight pound or touch and blood immediately goes out but dangerous because glass  (6 blank) |
| Uganda | the paper draws away blood immediately it's touched with the capillary  on slight pressing the blood flows out of it  blood easily flows without any pressure applied / no pressure applied, blood flows out by itself / blood flows out of the tube with ease; no pressure required / automatic, no manipulation required / it takes itself, you don't force it  you just put there, needs no energy, no squeezing, no work, it's automatic  easy to target in RDT, and blood flows easily, just place and blood can flow |
| **GLASS CAPILLARY, deposit: No, not easy**  Nigeria | because you will need to hit the capillary on the filter paper severally  several attempts of tapping is required to deposit blood  (NB all comments relate to difficult of depositing blood on relatively non-absorptive surface) |
| Philippines | difficult and takes time to be pounded out / you need to pound several times to release blood  (NB all comments relate to difficult of depositing blood on relatively non-absorptive surface)  (3 blank) |
| Uganda | it is too small / very small and difficult to see  does not release blood easily and the blood released is small / not all the blood comes out of it to the filter paper  no way one can force blood out of it, so blood takes time coming out  blood goes very slowly, needs to be faster (may go faster with RDT pad) / not on this paper, but may be easier on real RDT device / hard on paper, but may be easier on RDT / you have to tap it on paper to release (RDT may be different) / on that filter paper it was not easy, but maybe on the RDT it's easy  (NB most comments relate to difficult of depositing blood on relatively non-absorptive surface) |
| **GLASS CAPILLARY: Yes, risk of exposure**  Nigeria | blood spillage can occur during releasing of blood / during blood release  tendency to spill while tapping to release blood / tapping could cause splashing  (NB most comments relate to risk while depositing on relatively non-absorptive surface) |
| Philippines | easily broken / has a tendency to be broken  depends on the hands of the health worker, should be properly handled  has tendency to splatter  if not careful, blood can spill on health worker / if the patient is a child, blood might be spilled on him  (NB some comments relate to risk while depositing on relatively non-absorptive surface)  (3 blank) |
| Uganda | it is hard and can break easily / it's a brittle thing, so if it breaks for example when struggling with a child, it becomes a sharp / since it's glass, you might injure yourself or your pt as you fidget around so high risk of exposure, and may not burn after disposal  she felt that the blood could drop from the tube  if there is backflow of blood, it is risky then / due to back flow of the blood / open on both sides, blood easily comes out from the other end / open on both sides and blood can come out of the other [end]  it's short and so you can easily get in touch with blood / it's very short, blood could come out the top / because it's short, you work closely with the blood  if you're shaking, like I shake, you could easily expose yourself or the patient to blood  in case of children they may think it is a needle and they may struggle making it to break and injuring them  (NB a few comments relate to risk while depositing on relatively non-absorptive surface) |
| **GLASS CAPILLARY: No, not appropriate**  Nigeria | the application is difficult and it takes longer time to release  can only be used in laboratory  (NB most comments relate to difficulty, risk, time waste in depositing on non-absorptive surface)  (4 blank) |
| Philippines | it will be hard for BHWs to collect blood from patients using this device  easily broken and not safe to use / breakable, might be broken  blood does not enter immediately if only small amount is present  difficult to use and deposit / difficult to collect and pound out  (NB many comments relate to difficulty, risk, time waste in depositing on non-absorptive surface)  (6 blank) |
| Uganda | it takes a long time and it is small / it's tiny and may be difficult for people with sight problems  consumes time since it does things on its own pace / it takes much time to get the sample, when you are observing the blood  because if blood clots in it one may miss the right volume of blood  when the fingerprick blood is little, sufficient amount may not be obtained  because of risk of blood exposure / because of that risk (backflow of blood)  can cause injuries to patient and health worker, and disposal is rather hard (risky)  difficult to withdraw when wrongly positioned; releasing is also difficult  difficult for use with children because of time required to collect blood, time lag / it is not suitable for children and since being glass it may break / with fighting patients, like children or mentally disturbed, can't apply constant pressure to draw blood  risk factor of exposure and time wastage especially if patients are many  you will not feel comfortable with your client, because you prick much, the relationship is not good; also with children it takes time, they will be quarreling  (NB some comments relate to difficulty, risk, time waste in depositing on non-absorptive surface) |
| **CALIBRATED PIPETTE, collection: Yes, easy**  Nigeria | easy to manipulate pressure / easy to manipulate while collecting blood  with little pressure blood flows in / just little pressure is needed / once pressure is applied, blood flows in  although with tendency to overshoot mark / but with tendency to overshoot mark / although problem of overshooting mark often happens  except pressure manipulation may sometimes prove difficult  if the rules and procedures are followed it will be easy  (1 blank) |
| Philippines | easy to press and blood enters readily / blood goes with device fast once pressed / just press and blood enters immediately / just a slight pinch and blood goes immediately inside the device / by pressing, blood is suctioned  when controlled during pressing, blood goes in immediately / just control pressing of tube  slight press and release, blood goes in immediately  when pressed too much, blood goes up very high / blood goes up to the tip of suction if not controlled  not easy to control but fast in collecting  because it is small, short, a slight pinch can absorb/suction blood immediately  you can control and with one press, blood is absorbed  (19 blank) |
| Uganda | it sucks blood easily on squeezing / pressing is easy / on slight squeezing the blood flows in easily  easy to attain the blood at the required first mark / easy to attain the blood at the required first mark / it's marked, the health worker was aware of where to stop / it's marked and it was easy to balance pressure to get to the desired mark  you just press and it gives you the amount, you just have to observe  it was easy to estimate the desired volume of blood  she was experienced in using the calibrated pipette / she had ever used the calibrated pipette |
| **CALIBRATED PIPETTE, collection: No, not easy**  Nigeria | pressure is needed, it has to be done with effort  difficulty in controlling the pressure while collecting blood / difficulty in pressure manipulation  appropriate volume difficult to collect / a lot of pressure needed and the required volume may not be achieved  it takes too much blood / more than required blood is collected  pressure manipulating problem and tendency to overshoot mark / problem with pressure manipulation cause overshooting above mark / very difficult and problem with pressure manipulation causing overshooting above mark  because of the graduated line that has to be targeted / the graduated mark makes it difficult for blood collection  blood exposure can occur  (1 blank) |
| Philippines | hard to press, blood doesn't enter readily / difficult to press, blood is not suctioned immediately  difficulty in pressing, needs timing  difficult in collecting blood because it is difficult to control / pressing is hard to control  because it needs pinching, if not pressed, blood cannot be collected  difficult to press, blood might reach tip of suction and blood can't go down  difficult if not controlled; if patient is a kid may not be able to obtain blood |
| Uganda | the blood rushes upwards to the bulb when pressure is released / when you press, you might fill the whole of it with blood  may suck much blood on pressing (squeezing) / always picks much more blood than it is required / the blood is over-sucked beyond the required mark / manipulating pressure is difficult, hence pipetted sample passed mark / difficult to measure, may suck blood past mark / blood goes beyond 1st mark, it depends on how you hold it; tricky / blood always rushes past the marks  if you're not very particular, blood passes the mark, just like straw  it's difficult to get the appropriate volume of blood / because it was not easy to get blood up to the desired mark / difficult to control the amount of blood drawn to the mark level / estimating up to the desired mark was difficult  holding blood sample at the first mark difficult / holding the blood at mark is difficult / controlling and holding the blood at the first mark is difficult / it needs a lot of technique, requires pressure to be maintained  in pressing, difficult to remember when to press, it's confusing, blood came into bulb  you have to squeeze slowly as if you are taking BP, if you release quickly blood goes up to 3^rd^ line  you may apply much pressure, it needs when you press, you continue pressing; you can forget and press before you reach where you want to touch down, and blood splashes  it needed squeezing, when you’re dealing with a kid who’s disturbing you, you can easily release the pressure and the blood goes up  as I was trying to get blood, I have to be sure the blood did not go past 1^st^ mark, and my arm was shaking  needs accuracy; I could just press it, then releasing pressure, the blood goes up into this chamber here  blood sometimes exceeds the mark; when with a kid, you can fail, because kids are ever crying, to obtain this mark is not easy |
| **CALIBRATED PIPETTE, deposit: Yes, easy**  Nigeria | with applied pressure, blood is released / with little pressure, blood will be released / just with little effort, blood flows / easy and just requires little pressure to release blood  blood flows out easily  easy to manipulate pressure while releasing blood  easy to use and is not stressful  (7 blank) |
| Philippines | after pressing, blood is released immediately / once pressed, blood immediately goes with the device / blood is obtained after pressing / just a pinch and blood goes immediately with device / one press, transferred automatically  because it is short, blood easily comes out  easy to transfer, can transfer all blood  easy but if blood goes up too high, it cannot be transferred  (27 blank) |
| Uganda | just needed little pressure applied to drop away blood / all the blood was released on squeezing / releases blood easily on squeezing / blood is just a squeeze away from getting out of it / easy to apply pressure on the pipette and blood easily flows / only pressure application was required to expel blood / I knew it was automatic, just release pressure and blood comes out  bulb (dome-shaped thing) has bigger pressure to press out blood  doesn't take a lot of time  you press it straight, and it releases blood; easy because of point  once you put it on the target, whatever blood you have in goes to the target  was ever exposed and used it for long |
| **CALIBRATED PIPETTE, deposit: No, not easy**  Nigeria | because pressure allows blood to flow up and reduces volume of deposits  hard in texture therefore difficult to deposit  a lot of pressure must be applied / because it requires more pressure before it can release  entrance of air can disturb the release of blood on the paper / creates air bubble while releasing blood  it leads to spillage  pressure manipulation difficulty  (1 blank) |
| Philippines | slowly press since bubbles are produced when not careful  blood is scattered  difficult to press, it's hard  needs to be pressed a couple of times for blood to come out  difficult because it is being pressed / difficult because it is being pressed |
| Uganda | needs pressure of squeezing to be maintained to avoid blood from entering bulb  you need to be careful to release slowly, or you will leave blood inside  some blood was sucked beyond the mark hence forming bubbles  if blood has gone past 2^nd^ mark you press and it may drip in pieces  too much pressure may spill blood far away |
| **CALIBRATED PIPETTE: Yes, risk of exposure**  Nigeria | it can splash / it can splash if not careful  because of too much blood / too much blood is collected  pressure manipulation may cause spillage / pressure may cause blood exposure / pressure may cause spillage of blood / spillage tendency due to pressure manipulation / pressure was causing splashing of blood  during deposition |
| Philippines | if not carefully in collecting  (4 blank) |
| Uganda | you may release before you reach the spot where you want to release / she felt it was easy for her to squeeze the pipette before reaching filter paper / it can splash on you before you reach where you are aiming  since it requires squeezing the blood may easily drop when squeezed / much pressure may be applied on transfer thus risk of dropping it off  blood splashes when releasing after pressure has been applied  when you press, you suck the air; when you press using force, it can easily splash blood, so you need to be gentle  it's short; you can easily get in contact with blood / it's short, so easy to get blood on gloves or yourself / because it's short, you work closely with the blood  especially if you are not steady, as you fumble around the blood may splash  blood can splash, you might splash on yourself  forms bubbles that spill blood in the surrounding  you’re trying to get blood exactly at the mark, and if the child is shaking, you risk splashing  (1 blank) |
| **CALIBRATED PIPETTE: No, not appropriate**  Nigeria | it takes more than the required blood / due to more volume of blood being sucked  because of overshooting and volume of blood is not usually accurate / required volume of blood may not be achieved  not accurate and difficult to manipulate  wastes time and not easy to manipulate  it takes time and not very accurate while collecting blood  not user friendly and difficult to manipulate / due to the technical capacities needed  because of the difficulty in collecting blood  sucking and releasing not user friendly / problem of pressure affects performance, pressing and releasing problems / problem of manipulating pressure / pressure does not make it user friendly  you draw more than necessary, it tends to retain blood; tend to also over- or under-release  difficult to release blood although easy to collect  it splashes blood and not easy to use  because it has tendency of spillage when depositing blood  due to risk of spillage, it is very dangerous  (3 blank) |
| Philippines | blood bubbles are produced, not good in placing blood  entrance of blood is hard to control / hard time in suctioning blood  difficult to press and let blood go out  difficult to control / should have control in using device  difficult to press and let blood go out  (3 blank) |
| Uganda | it may easily get much blood  hard to maintain the pressure  not easy to regulate the required amount of blood / not easy to draw the required amount of blood / it is very hard to measure the correct amount  it is difficult to obtain blood up to the desired mark  there are high chances of blood going up to the bulb of the pipette / when you press, blood may come into bulb and it remains with blood in it, not accurate  size of drop not uniform, because blood sucks into bulb and can't push it out; measuring is still a problem  by fidgeting w collection pressure, patient would even lose confidence in health worker  blood can go past mark; it's short, so easy to get blood exposure  exposure risk as a result of splashing when blood is deposited on filter paper  requires a lot of skills and careful handling / somehow tricky, may not give accurate results, needs a lot of practice  time wasting, needs enough expertise and anticipates risk  takes time to tap blood, not safe / very slow and needs a lot of accuracy  risk of contamination  takes time to estimate the amount of blood; needs one to be stable and risk of exposure as a result of bubbles  if the health worker is shaking, it may easily release the pressure; for adults patients it’s OK, but with kids, when they are disturbing, it’s not OK  no, because of delays with collecting blood; if you use it in your daily work, it will delay you  it takes much time; if you have many patients and your arm is shaking on one patient, when will you finish?  (1 blank) |
| **CUP, collection: Yes, easy**  Nigeria | it is just a touch and required amount is collected / just single touch needed / it is very easy, just single touch needed / just a dip is needed / very easy to manipulate, it requires only a touch  it picks amount of blood required / it takes required amount of blood / it collects the required volume  easy to manipulate it only requires a touch  very easy to handle / very, very easy to manipulate device / very, very easy to use  easy, does not waste time / it is faster  the easiest of all devices  no effort needed |
| Philippines | fast to collect  after touching the fingers, blood immediately goes with the device / with a press of the fingers, blood goes immediately with the device / just press on fingers and blood goes in immediately  just one touch, blood is immediately obtained  it is just placed beside blood and it is already absorbed / just put close to blood and it goes with the device  easy in terms of collecting, easy to obtain because blood goes immediately with it / easy to use in collecting, just place it slightly and it can already collect  blood obtained was just enough  easily but only few blood is collected  (22 blank) |
| Uganda | it's open and blood just enters when touched / requires touching only / just a matter of placing it on the sample, blood fills in automatically / collects blood by itself when in contact with blood / it picks blood on touch / requires only a single touch / just dipping in blood was enough to pick the blood  removes blood faster with a single touch  it has a depression on top for easy collection with a single touch  it did not require any squeezing; it was a matter of touching blood / it picks blood by itself without any pressure / just a matter of pointing into blood and it picks enough without pressing or forcing / taps blood by itself with no pressure applied or any manipulation  a single touch obtained blood; easy to use and saved time  long enough; no pressure applied, no measuring the amount of blood  easy, faster, understood after a very short time  didn't require any training on how to use it; also time-saving  easiest, it's automatic  just a moment of touching on the blood and it comes on it; most appropriate one  just a touch with very little attention and blood fills  has its own measurement and fills by itself very quickly |
| **CUP, collection: No, not easy**  Nigeria | (no “no” answers) |
| Philippines | can collect blood right away when placed to finger but can get small amount only  small amount of blood obtained and has tendency to be spilled  blood doesn't go readily with the device/can't pick up blood / blood has difficulty going with the device / blood does not go with the device after touching it / blood does not go in immediately  not sure if blood immediately went with the device  difficult in collecting blood, not all goes in  (1 blank) |
| Uganda | the position of the tip is not appropriate for collecting blood  I was inserting all of device into blood, and it took too much |
| **CUP, deposit: Yes, easy**  Nigeria | easy to manipulate while depositing blood / it releases easily / easy to use and device is not stressful  easy to use and doesn't waste time / easy to release, no time wasted / does not waste time  very easy, no expertise needed  as soon as the cup touches the place of deposition, it is released / when you touch the paper the blood is released  easy to release, no need to apply pressure / very easy to release and requires no pressure / very easy to use and requires no pressure  just a touch is needed |
| Philippines | fast in releasing blood  easy to place, just needs to be pounded out on the filter paper / easily pounded out, transfers by itself / just place on blood and it will immediately transfer  it is flat, just touch w the filter paper  just touch to filter paper and blood is immediately placed to it  (26 blank) |
| Uganda | blood spreads away on the filter paper immediately it is touched / blood spreads out to the paper automatically  touching the filter paper causes release of the blood / drop immediately touches and blood sucks into absorbing surface  does not require pressing / it doesn't need squeezing since a single touch is enough / you just put, don't have to apply any pressure or do anything, just put on the paper/surface / put at surface, no need to squeeze / needs no special technique since the blood comes out on touch  it doesn't take time since it needs only a single touch / doesn't take time / also time saving, blood absorbs faster from the cup  because it has no tube where the blood would hide  the tip is straight for easy release of blood  you have to shift it, needs some technique, easy after a few practices / just needed you pressing it and rocking or pushing a bit  the amount picked gets absorbed, the whole of it  I pointed it onto paper which sucked all blood without any risk  needed no pressing; just touching paper vertically, blood comes out  100% easy because it releases blood when touched on the paper |
| **CUP, deposit: No, not easy**  Nigeria | (no “no” answers) |
| Philippines | small amount of blood placed / small amount of blood collected  difficult to place blood, should be pressed to remove blood  blood takes time to consume, not collected immediately  some are left, sticking in the cup / not all blood is transferred, some are left in the cup  (3 blank) |
| Uganda | does not release all the blood / not all the sample was absorbed by the filter paper / it held some or most of my blood sample, didn't release all  it needs some technique, you must rock it on surface before it releases |
| **CUP: Yes, risk of exposure**  Nigeria | (no “no” answers) |
| Philippines | it would drip if not done slowly  if the patients are kids, it can easily spill  has tendency to be spilled when hands of BHW are shaky  not safe in terms of being spilled because it is open / it is open with a possibility to spread  (4 blank) |
| Uganda | because the blood can also touch the outside parts of the cup  if you are dealing with kids, in case he's fighting w you, the blood could fall off |
| **CUP: No, not appropriate**  Nigeria | (no “no” answers) |
| Philippines | it would drip and spill and in terms of ease of collection, it is difficult  blood does not immediately go with the device / difficult in collecting blood, especially if there are many patients / blood does not go in, difficult to collect  has difficulty, it might spill especially if patient is moving  small amount of blood obtained / small amount of blood is obtained  blood is difficult to remove  (1 blank) |
| Uganda | took too much blood, then held some of sample and didn't release all  because of risk of blood exposure as example with struggling child |
